# The Doctors, Their Patients, and the Symptom Checker App: Qualitative Interview Study With General Practitioners in Germany

**DOI:** 10.2196/57360

**Published:** 2024-11-18

**Authors:** Christine Preiser, Natalia Radionova, Eylem Ög, Roland Koch, Malte Klemmt, Regina Müller, Robert Ranisch, Stefanie Joos, Monika A Rieger

**Affiliations:** 1 Institute of Occupational and Social Medicine and Health Services Research University Hospital Tübingen Tübingen Germany; 2 Institute for General Practice and Interprofessional Care University Hospital Tübingen Tübingen Germany; 3 Institute for General Practice and Palliative Care Hannover Medical School Hannover Germany; 4 Institute of Philosophy University Bremen Bremen Germany; 5 Faculty of Health Sciences Brandenburg University of Potsdam Potsdam Germany

**Keywords:** symptom checker app, qualitative interviews, general practice, perceived work-related psychosocial stress, job satisfaction, professional identity, medical diagnosis

## Abstract

**Background:**

Symptom checkers are designed for laypeople and promise to provide a preliminary diagnosis, a sense of urgency, and a suggested course of action.

**Objective:**

We used the international symptom checker app (SCA) Ada App as an example to answer the following question: How do general practitioners (GPs) experience the SCA in relation to the macro, meso, and micro level of their daily work, and how does this interact with work-related psychosocial resources and demands?

**Methods:**

We conducted 8 semistructured interviews with GPs in Germany between December 2020 and February 2022. We analyzed the data using the integrative basic method, an interpretative-reconstructive method, to identify core themes and modes of thematization.

**Results:**

Although most GPs in this study were open to digitization in health care and their practice, only one was familiar with the SCA. GPs considered the SCA as part of the “unorganized stage” of patients’ searching about their conditions. Some preferred it to popular search engines. They considered it relevant to their work as soon as the SCA would influence patients’ decisions to see a doctor. Some wanted to see the results of the SCA in advance in order to decide on the patient’s next steps. GPs described the diagnostic process as guided by shared decision-making, with the GP taking the lead and the patient deciding. They saw diagnosis as an act of making sense of data, which the SCA would not be able to do, despite the huge amounts of data.

**Conclusions:**

GPs took a techno-pragmatic view of SCA. They operate in a health care system of increasing scarcity. They saw the SCA as a potential work-related resource if it helped them to reduce administrative tasks and unnecessary patient contacts. The SCA was seen as a potential work-related demand if it increased workload, for example, if it increased patients’ anxiety, was too risk-averse, or made patients more insistent on their own opinions.

## Introduction

The right to diagnose is reserved for physicians and is a core element of professional authority [1]. Finding the proper diagnosis as part of GPs’ expertise has been reported to contribute to physicians’ perceived job satisfaction [2]. Patients also play an important role in the process. They first assess their symptoms, schedule an appointment, present their symptoms, and react to the diagnosis [3,4].

But what happens when technologies enter this process? Here we explore this with the example of the perspectives of general practitioners (GPs) on the Ada App, an internationally available symptom checker app (SCA).

Since the 1950s, physicians and computer scientists have been exploring how computers can be used to assist and improve the diagnostic process [5,6]. Part of the technologies are primarily designed to assist trained physicians or other health care professionals. Other technologies, such as symptom checkers, are designed for laypersons. A plethora of different apps exist [7]. The wording about what browser- and app-based symptom checkers actually deliver is rather heterogeneous, as the debate is ongoing [8]. For the purpose of this study, we use an operational definition: symptom checkers provide lay users with preliminary diagnoses, give a first sense of urgency, and suggest a course of action [9]. As such, we understand symptom checkers as “sociocultural artifacts” that are “(nonhuman) participants in networks of meaning and power relations” [10].

Symptom checkers are discussed ambivalently in the literature. Some expect that symptom checkers could outperform physicians’ diagnostic capabilities [11], increase anxieties in patients [12], disrupt the doctor-patient relationship [10,13-15], or cause overuse of the health care system [16,17]. Further perspectives suggest that physicians’ expertise and authority remain unchallenged by symptom checkers [10,18,19], patients could be empowered [12,18], and symptom checkers might have the potential to reduce physicians’ workload and relieve an overburdened health care system [15,20,21]. Therefore, despite physicians not being the primary target group, symptom checkers might have far-reaching impacts on their work in terms of work content and work organization. These two dimensions are central to established models of perceived work-related stress [22-25].

As GPs are the primary access point to the health care system for patients [26], they might be particularly affected by laypersons’ respectively patients’ use of symptom checkers and the aforementioned impacts such as the use of the health care system or changes in the doctor-patient relationship. To date, little is known about the lived experiences and perspectives of GPs in regard to SCAs. A survey among GPs in the United States showed that only 30% had first-hand experiences with chatbots in health care [27]. One survey from Finland analyzed the experiences of health care professionals with a symptom checker at occupational health clinics and found that symptom checkers were of limited relevance in their daily work [15]. In both studies, the attitudes of the respondents mirrored the aforementioned discourse. While the studies give a first overview of relevant topics in the field, there is no in-depth analysis of how GPs experience SCAs as nonhuman participants in their daily work and how they see SCAs in relation to their own “apostolic function” [4], that is, their expectations on patients, their illness concepts, and personal and professional values and attitudes. Depending on how GPs disintegrate or integrate SCAs in their “apostolic function,” this, too, might have further impacts in regard to job satisfaction and perceived work-related stress.

This study is embedded in the multidisciplinary joint project CHECK.APP, in which the ethical, legal, and social implications of SCAs in general practice in Germany are analyzed using the example of the Ada App [28]. The Ada App [29] had originally been conceptualized as a clinical decision support system but was later redesigned for laypersons. Laypersons enter their symptoms guided by the questions in the app and will be presented with a ranking of several potential diseases in combination with a sense of urgency and potential courses of action. It appeared to be a promising case for a wider phenomenon. Its advertising campaign #tellada won the German brand award in 2019 and included slogans such as “How are you? Be honest” [30,31]. At the same time, the actual number of users remained unclear. A health insurance had planned to implement the app, but it canceled its plans due to data privacy concerns [32-34]. As such, the app represents the aforementioned ambivalence around SCAs. In this study, we take a technology-in-practice approach [35] and will focus on GPs’ lived experiences with the SCA and their perspectives on it. From an occupational health perspective, we are particularly interested in the following research questions: (1) How do GPs experience the SCA in relation to the macro, meso, and micro levels of their daily work? (2) How does this relate to their professional identity, job satisfaction, and psychosocial resources and demands?

## Methods

### Study Design

In a scoping review on the impacts of laypersons’ use of SCAs on physicians in primary care, we showed that while some publications include the perspectives and voices of physicians, they primarily portray expectations rather than lived experiences [36]. In this empirical study, we aimed to fill this gap by exploring the experiences of GPs in Germany through the in-depth reconstructive analysis of semistructured qualitative interviews. The interview study was embedded in the joint project CHECK.APP which integrates the perspectives of users, experts, and GPs and works with a mixed methods research design, including a scoping review on ethical, legal, and social aspects [8], a survey among the wider German population, a diary and interview study with app users, qualitative interviews with GPs, and qualitative interviews with experts with regards to SCAs in general and the German health care system in particular [28].

### Sample

We used several common recruitment strategies: as we know from previous studies, it is almost impossible to contact GPs directly by telephone, as practice assistants act as gatekeepers [37,38]. We contacted GPs by letter, email, or fax, depending on the information available through internet searches, and sent follow-up messages after 2-4 weeks. In addition, one of the GPs in the collaborative project (RK), who was not directly involved in this part of the study, contacted GPs through his institute’s practice network. Our final sample included 8 GPs from different regions of Germany, representing different theoretically derived dimensions of our intended sample (Table 1).

**Table 1:** Sample of the study.

Relative dimensions and feature				Value (N=8), n
**GPs^a^**				
	**Age (in years)**			
		<45	3	
		45-55	2	
		55+	3	
	**Gender**			
		Women	3	
		Men	5	
		Diverse	—^b^	
	**Race**			
		Black	—	
		Person of color	—	
		White	8	
	**Migrant**			
		Yes	1	
		No	7	
	**Experiences with SCA^c^**			
		Yes	1	
		No	7	
	**Use of digital tools**			
		No indicators	2	
		Indicators such as browser- or app-based system to book appointments, telemedical appointments, focus of the practice, and profile in social media	6	
**Practice**				
	**Location**			
		Rural	3	
		Suburban	3	
		City	2	
	**Structure of the practice**			
		Single practice	3	
		Joint practice	5	
		Medical center	—	
	**Size of the practice**			
		<5 employees	3	
		>5 employees	5	

^a^GP: general practitioner.

^b^Indicate the dimensions that, despite our efforts, were not represented in our sample.

^c^SCA: symptom checker app.

### Data Collection

We developed the interview guide in several rounds, translating our research interests into final interview questions [39] (Multimedia Appendix 1). Two researchers (NR and CP) and one research assistant (EÖ) each conducted at least one of the interviews. Each interviewer obtained verbal and written consent for their interview. All 3 interviewers were trained sociologists with 3 (EÖ), 6 (NR), and 10 (CP) years of professional experience in health services research and occupational medicine. The interviews were conducted between December 2020 and February 2022 using the video conferencing tool VidyoConnect (version 21.6.3.17468; Vidyo Inc), which is provided by the University Hospital of Tübingen. The interviews lasted between 30 and 63 minutes and were recorded using an external audio recorder. The videos were not recorded. The files were transcribed by a certified office and pseudonymized by NR.

### Data Analysis

The interviews were analyzed by NR, EÖ, and CP using the integrative basic method [40]. This reconstructive-interpretive method allowed the reconstruction of manifest and latent meanings by analyzing semantics, syntax, and metaphors. We chose agency and positioning as analytical approaches [40] because these proved particularly promising for understanding how GPs see and navigate themselves and their agency in a network of potentially conflicting participants and interests. We used the “Risk assessment of work-related psychological stress” of the Joint German Occupational Safety and Health Strategy (Gemeinsame Deutsche Arbeitsschutzstrategie) which operationalizes established models of work-related perceived stress [41] as a core sensitizing concept [42] for our analysis. It defines work content, social relations, work organization, work environment, and new forms of work as dimensions of work-related stress.

All interviews were interpreted line by line in order to identify the main motives and modes of thematization. Analytical case protocols were written for each interview. Interviews were continuously compared with each other. For the purpose of quality assurance, the analyses were discussed with MAR and researchers of the joint project [40]. Furthermore, we conducted a member check [43] with study participants and experts on SCAs in Germany in April 2022. The reporting of this study follows the Standards for Reporting Qualitative Research guidelines [44].

### Ethical Considerations

The ethics committee of the Medical Faculty and University Hospital of Tübingen has approved the study (464/2020BO). All study participants were informed and gave consent verbally and in written form.

## Results

### Overview

The results reflect the perspectives of GPs and are presented along the main stages of the diagnosis process: the unorganized stage, the patient’s decision to see a GP, and the shared process of exploring the patient’s condition.

### Doctor, Have You Heard of Ada?

The GPs in this study were mostly receptive to digitalization in their area of practice and used digital tools to varying degrees to manage patient volumes and streamline workflows. These included tools that helped to free up the telephone line, tools that helped with documentation, and tools that enabled easy and direct digital communication between patients and the practice. Several GPs made it clear that the digital tools were not suitable for all patient groups but helped to relieve capacity for those patients who did not use digital tools (eg, ensuring telephone availability). Although the GPs in this study were rather receptive to digital tools, only one GP was aware of symptom checkers in general and the introduced SCA in particular. This GP used it as an additional interlocutor:

GP 3: (2) So I personally use it for patients with … rare symptoms or unusual laboratory constellations ... too. So then ... I ask Ada, ... simply to get differential diagnoses again and then think about it: Could any of these differential diagnoses be correct?

Interviewer: During the consultation?

GP 3: No, in the evening on the couch (laughs).

Interviewer: (laughs) So you don’t finish work, but still google about Ada in the evening ... another symptom?

GP 3: Exactly. So that’s ... I usually already have an idea, and if it’s a more complicated case, I also take a blood sample. And the blood values ... I go through them in the evening anyway. And if I then somehow come to a standstill or think maybe I’ve forgotten something in the differential diagnosis, then I often use Ada and ... see if it gives me new ideas, new impulses, yes.

female, <45 years old, rural area

This means that our results reflect expectations rather than experiences of the specific SCA and SCAs in general. However, the differences between GPs who were familiar with the SCA and those who were not were mainly in aspects of the app’s practicality, not in perspectives on the app or their work.

### The SCA in the Black Box of the “Unorganized Stage”

GPs normalized patients’ desire to explore their condition and considered it helpful if patients had already thought about their symptoms, as this might help some patients to accept the outcome of the consultation.

So there is the example of the well-informed, intelligent patient who has already obtained preliminary information, which may not always be correct, but which sometimes makes it more difficult to find a diagnosis because it is unfiltered information. But I would say that, looking at all patients, it is easier if the patients have been informed in advance. You often have to revise patients’ misjudgements, but they have already considered the issue in more detail. That is on average ... It certainly always depends on the type of patient you have in the practice, but ultimately it helps rather than harms if the patients are preinformed.

GP 8, male, 55+ years old, big city

At best, the SCA could help patients reflect on their condition in preparation for a consultation with their GP, or reduce unnecessary patient anxiety. However, patients may include irrelevant information or omit relevant information, leading to misleading results. GPs portrayed patients as laypeople who often focus on and present subordinate aspects, use the wrong search terms when searching the internet, or are unable to assess the quality of information and follow the most appropriate course of action. According to GPs, internet searches in particular can increase patients’ uncertainty and anxiety, as they may experience a flood of (negative) information and focus on the most serious potential outcomes. Some GPs preferred the SCA to internet searches because the information might be more evidence-based and focused. As such, GPs did not consider the SCA to be suitable for all patients, but only for those with eHealth literacy, general health literacy, and anxious patients, for whom the SCA might be the lesser of two evils compared to internet searches.

(...) then the app might come up with an initial result that is not quite as bad as a search engine. I don’t know whether this will reassure patients, because someone who is worried about their health from home doesn’t know whether they’ll trust the app’s result, and in the end the app has to present the result, so if in doubt, they’ll go to their doctor. If one or two people with a cold don’t rush straight to the GP’s surgery, then perhaps there could be a marginal relief effect for GP surgeries...

GP 7, male, 45-55 years old, suburban area

However, GPs mostly portrayed patients’ path to information as a black box for GPs, as patients did not necessarily disclose their sources of information. It became clear that GPs also rarely actively asked patients where they got their information from, as they considered the source of information to be secondary once patients were in the consultation room. GPs therefore focused on what they could control: direct contact and discussion with patients. In addition, GPs emphasized that asking about the source of information would in most cases take up scarce time without adding value to their work. They felt it was more important to probe the patient’s understanding of their condition (“disease model” [German: Krankheitsmodell]).

### (Better) Too Early, (Than) Too Late—The SCA and Patients’ Timing

GPs expressed that they were dependent on patients’ decisions about when to see a doctor. They expected patients to go to the GP at the right time, but in their narratives, patients often went either too early or too late. This reasoning was embedded in the GPs’ understanding of health care as a system with limited resources, with the scarcest resource being health professionals’ time. In all interviews, GPs referred to the context of their working conditions, which may also shape their expectations of SCA. All GPs reported an intense workload with a high number of patient contacts. The workload has been increased by the COVID-19 pandemic. When in doubt, GPs preferred patients to seek help too early to avoid avoidable suffering. For them, the most important question about the SCA was: would it encourage patients to seek help at the right time? If it was too risk-averse, it would send patients too early and lead to oversupply, sabotaging the GP’s mandate.

So, the back pain doesn’t have to be something bad, you’re doing this and this and this and we’ll talk again in a fortnight and you’ll tell me how it’s gone then. So a watchful waiting approach. Whereas so-called red flags in general medicine, that is, obviously highly conspicuous and potentially dangerous symptom indications, lead to immediate consequences. This is how we handle evidence-based general medicine, at least in my practice. If the Ada app now turns everything into red flags ... it not only destroys our health care system but also unsettles patients and does the opposite of what is also very important in general practice, namely the prevention of overdiagnosis. For a variety of reasons, of course also for reasons of cost, the economic costs, but also (pauses) to ... yes, to keep the feeling of illness away from patients.

GP 5, male, 45-55 years old, rural area

Conversely, if the SCA was not risk-averse enough, it would lead to underuse for patients. GPs attributed the potential for both to the SCA. The SCA could give wrong results and exacerbate the challenge of resource scarcity, or it could give patients the right sense of urgency and self-care instructions for simple cases. However, GPs felt that this risk or potential of the SCA would ultimately depend on the competence or personality of the user, not the technology itself.

Some GPs were considering how to integrate the SCA into their workflow, particularly with regard to practicalities that might have the potential to reduce unnecessary patient contact and thus workload. Some imagined that the results could be made available to GPs in advance.

So ultimately it’s just a decision: He has to come or he doesn’t have to come, or I have to visit him or not visit him. Those are the two things that are ultimately at the end of the decision-making tree. Yes. And I have to make the decision in the end when I have looked at it and realize: OK, it doesn’t look very good somehow. Then you just have to say: You have to come. Or you just have to say: I’m going there. So, that ... (2) But you don’t have to ask about all this previous history because it’s already done. And, well, you have to be able to rely on it, that’s the crucial thing (laughs).

GP 4, male, 55+ years old, suburban area

In this way, the SCA would document the initial history and become a tool for communication between the patient and the GP, while the GP would ultimately decide how to proceed.

### Finding Diagnosis—Humble Paternalism and the Art of Sense-Making

GPs described finding a diagnosis as a process that could involve several steps, patient contacts, and time (watchful waiting, see also the response of GP 5 above). From the GPs’ perspective, both GPs and patients had a common goal: to find out what the patient had and to take the necessary measures. GPs and patients also shared the challenge of limited knowledge (see also the response of GP 3). However, from the GPs’ perspective, GPs as medical experts and patients as medical laypeople had different knowledge limitations and different roles in the process. In the interviews, GPs positioned themselves as experts who, as such, were better able to search for information and assess information quality (including internet searches in medical databases and popular search engines), given their existing broad medical knowledge and years of professional experience. GPs positioned themselves as the medical authority who knew better, but who also had an ethical and legal responsibility to do their best to reach the right conclusion and treatment. GPs expected trust from their patients and offered skepticism in return. In the diagnostic process, they expected patients to share (information relevant to the GP) but not to overshare (information irrelevant to the GP) and thus to contribute smoothly to the GP’s work.

So my favorite patient is the one who describes their complaints and not immediately their interpretation: “I read on Google, and that fits together.” (laughs) “No, stop, (laughs) that’s what I do (laughs). I would need your complaints (2) and not your interpretation of your complaints, please leave that to me.” Hm, well, I would say about 20% of patients already prepare information from the internet. But to be honest, that bothers me more than it helps.

GP 6, female, 55+ years old, big city

The GPs in this study reacted negatively to terms such as “self-diagnosis,” which we used in the interview guide in reference to common wording in the literature on SCA. Our interviewees rarely used the term “diagnosis” themselves. Rather, they spoke of “ideas,” “assessments,” “interpretations,” “perceptions,” “categorizations,” etc in relation to SCA, but also in relation to their work. GPs considered it potentially helpful for patients to gain an initial understanding of their case, as long as they remained open to the GPs’ guidance. They therefore describe the process of exploration as one of guided shared decision-making, with the GP guiding the patient and knowing best, but ultimately knowing that the patient will decide their own direction.

When it came to the SCA in this process, the GPs drew clear boundaries.

But yes, I actually see myself more as a symptom checker myself, so (laughs) people come to me and tell me their symptoms and I’m the one who helps them categorize them. (...) I think I can do that better than any app (laughs).

GP 1, female, <45 years old, rural area

The GPs ascribed to the SCA the potential to act as a nonhuman actor. The SCA could help guide the process of exploration—but it could also be an uncontrollable element that “spits out diagnoses,” as one GP stated. GPs problematized the SCA if it disrupted their workflow and the doctor-patient relationship. GPs were concerned that the SCA could create avoidable extra work over which GPs had no control if GPs had to deal with the impact of the app on patients before they could focus on their work. The SCA was seen as an intruder if it cemented patients’ insistence on their ideas and lay diagnoses, thus causing extra work:

So I think that if the app ... for example, really just says: “Go to your GP at short notice, he should clarify this,” I have no problem with that at all, I think that’s a great thing. Then I don’t have to do a lot of educational work, I can look at the patient and then make a decision and discuss it with them. However, if the app now throws specific diagnoses into the room, and that’s what it does here, i.e. infection with the bacterium Clostridium difficile, then it could be that the app leads to more work for me because then I first have to work through the app’s diagnoses and reassure the patient, and above all I have to justify why I think this is not the right thing to do, even though the app suggests it. (...) I would find that unfortunate.

GP 2, male, <45 years old, suburban area

The GPs positioned themselves as the real intelligence against the artificial intelligence (AI) behind the SCA. From their point of view, the SCA could not offer anything that a human could not: the SCA had no empathy and could not offer physical examinations. GPs could send patients to a specialist when they reached the limits of their expertise, while the app would not send users to another, more appropriate app. They made it clear that the diagnostic process is more complex than a compilation of symptoms. From the GPs’ perspective, diagnosing and finding the line between ill and not ill was a process of making sense of information. The SCA could collect potentially relevant information, and at best, organize it, but would not be able to make sense of it. GPs presented their view as limited but more objective, neutral, and less biased than that of the SCA, whose results were seen as one-dimensional. Ultimately, GPs concluded:

That depends on what the take-home message is for the patient at the end of the AI utilization, as I just said. If the take-home message is: You have complaints that (3) ... should result in a doctor’s consultation within the next week - then I think that’s fine. (00:30:55) If it’s different, (...), then I would say to the patient: OK, now ... we’re starting from scratch, please don’t tell me anything about Ada, but tell me everything you told Ada again because I’m not going to let an AI take my decision-making away from me. If I go through a PHQ-9 questionnaire ... with my patient who has depression, then the questionnaire doesn’t make the diagnosis, I do. But it can contribute to the validation of the diagnosis, in addition to my medical skills. And that’s why AI can be part of the doctor-patient relationship, but in my view, it can’t replace it - at least in the GP sector, where it’s all about relationships.

GP 5, male, 45-55 years old, rural area

## Discussion

### SCAs as a “Proximate Future”

This study is one of the first empirical studies of GPs’ perceptions of SCA in their daily work. One of our main findings is that SCAs seem to be much less relevant in the current daily work of GPs in Germany than we had initially expected from the scientific literature and public discourse on symptom checkers. There may be several reasons for this. As we found out in the joint project, the use of symptom checkers is not widespread in the German population, that is, only a small proportion of patients actually use symptom checkers [45]. Furthermore, patients have been shown to be reluctant to share their source of information with physicians for fear of criticism from their physicians and disruption of the physician-patient relationship [46], so even if patients are using the particular SCA or other symptom checkers, they are unlikely to tell their GPs. In addition, GPs in this study reported that they did not ask patients about potential sources of information and did not differentiate between digital sources, but used the umbrella term “internet searches” for all types of digital tools. GPs might consequently have experiences with patients who use symptom checkers but might not be aware of this experience. To date, symptom checkers are rather a “proximate future” in German health care, a yet unachieved future envisioned by tech companies or other stakeholders as to be “just around the corner” and to be about to solve pressing issues of the present [47]. Centering “proximate futures” and the associated techno-utopian or techno-dystopian visions tend to distract from the unresolved issues of the present [47]. For us, then, the question is what we learn about the present work of GPs through the lens of GPs’ perceptions of symptom checkers.

### GPs’ Perception of the SCA in Their Daily Work

GPs used language that presented the SCA and symptom checkers as “(nonhuman) participants in networks of meaning and power relations” [10]. Our results address SCA in GPs’ perspectives in relation to the macro, meso, and micro levels of their work.

At the macro level of the German health care system, we are to date not only facing a demographic change in the German population and among GPs (in 2022, about 36.6% of the GPs in Germany were 60 years or older [48]) but also a shortage of GPs due to lack of young practitioners, physicians’ wish for more part-time work, and an uneven distribution between urban and rural, as well as high- and low-income areas [49,50]. This is prognosed to intensify within the next decade. Some authors envision symptom checkers or other AI- or algorithm-based technologies as promising tools in a health care system of increased scarcity [17,51]. At present, symptom checkers are hardly used among the population in Germany [45,52] and—as shown by our data—are hardly known among GPs. GPs were critical about the future potentials of symptom checkers as useful participants in the German health care system if symptom checkers were too risk-adverse or not risk-adverse enough and led patients to the medically wrong time within the health care system. Patients’ individual decisions to follow the suggestions of the SCA might thus have impacts on the macro level and lead to over- or undersupply. Nevertheless, patients’ current decision-making process is more complex than following the results of a symptom checker [52,53].

At the meso level, GPs considered the SCA as a nonhuman participant on the organizational level of the practice and the interpersonal level of the physician-patient relationship. Digitization in the German health care system is a fragmented and slow process [54,55]. On an organizational level, GPs work in an environment that is characterized by a patchwork of technologies that lack interoperability. They perceived symptom checkers to feed into this pattern instead of improving it. Some GPs currently use digital tools to streamline patient contacts and administrative tasks. GPs welcomed the SCA as a potential future technology if it facilitated documentation and administration or helped to reduce unnecessary patient visits. In regards to the physician-patient relationship, GPs located the SCA in the black box of the patient’s search as part of the patient's “unorganized stage” [4] and “prediagnosis work” of patients [3], where it might complicate but not fundamentally damage the GPs’ and patients’ shared process of exploration. The SCA was attributed to the larger phenomenon of internet searches and “Dr Google” [56] which does not create a new phenomenon, but at worst reinforces an already existing one, namely patients who distrust their own health literacy, anxious patients, or patients who insist on their own assessment instead of trusting that of the GP. However, as the SCA is hardly present in the GPs’ daily work, it is not perceived as having a real impact on the meso level.

On the micro level, GPs positioned the SCA in relation to themselves. GPs in this study reacted negatively to terms such as “self-diagnosis,” often used in the popular and scientific literature on symptom checkers [16,46,57], and rejected the idea that the SCA could provide a proper diagnosis. They saw the SCA as lacking the holistic view, empathy, accumulated experience, and flexibility of human physicians ([58]). Similar to other studies, GPs saw diagnosis as a process of making sense of data and information and understood the SCA as monodimensional and static, full of correct information but unable to make full sense of the data (cf. [58]). They saw themselves as real, complex, and flexible, adaptive intelligence, acknowledging their own biases and knowledge limitations, compared to an artificial, schematic thinking intelligence ([10,20,58]). They therefore framed medicine as science and art, and technology as data minus art in the present and the future. As such, the SCA would not touch the GP’s professional expertise. This perspective also resonates with the current quality of symptom checkers. Databases in medicine might grow, but are biased and reinforce inequalities, and health data is of economic and political interest [59-62]. When it comes to SCAs, currently, the diagnostic accuracy of symptom checkers does not match the vision [63-65] even after decades of research [6,7,66], but they have the potential to provide an initial sense of urgency [17,63,64].

### The SCA in Relation to Perceived Work-Related Resources and Demands

From an occupational health perspective, we were particularly interested in what our findings mean in the context of professional identity, subjective job satisfaction, work content, work organization, social relationships, and work-related psychosocial resources and demands [67]. The focus on the SCA in the following should not obscure the fact that the causes of the main work-related stressors for GPs can be found and should be addressed at the macro level [68,69].

Studies have repeatedly shown that GPs in Germany have a high workload, long working hours, and a higher prevalence of burnout [70,71]. The GPs in this study are no exception to this and faced an even increased workload due to the COVID-19 pandemic. Lack of enough time is a key stressor for GPs. As a consequence, their perspective on both patients and the SCA alike is structured by the question: Who or what will cost time for what? GPs see some aspects of the SCA as a potential resource in their work. Similar to other publications, the SCA is seen as a better source of health information for patients using internet searches than, for example, popular search engines or digital encyclopedias ([12,46]). The SCA is seen as potentially increasing work demands if it increases patients’ anxiety, is too risk-averse and sends patients to the doctor unnecessarily or makes patients more insistent on their view, that is, if the effects of the SCA add to the already high workload or interfere with the doctor-patient relationship as an important part of GPs’ work. GPs also see the SCA as a potential resource if it “streamlines access to physicians” [58], for example, if they can see and work with the results and inform patients whether or not they should come to the practice ([17]). The latter aspect has implications for the organization of work of GPs and possibly the practice team, for example, the question of when and how to integrate the results of the SCA into the workflow [72]. A study of a symptom checker embedded in an occupational health clinic shows that physicians do not integrate the results into their workflow [15]. As implementation theories and patient-reported outcome studies have shown, the additional information will only be used by health care professionals if it is meaningfully embedded into workflows [73-75]. In another recent study, the use of an AI-based chatbot did not reduce the workload of GPs or the duration of patient visits [76]. Furthermore, if the integration of SCAs leads to an even higher density of patient-related decisions to be made by the GP or the practice team, or if they are dealt with during breaks, the potential resource could become a demand.

For the GPs in this study, similar to other studies, patient work and especially the doctor-patient relationship is an important resource in terms of their subjective job satisfaction [68], as well as finding the proper diagnosis [2]. It has been shown that administrative tasks are the least favored tasks and inhibit job satisfaction [2,77]. Anything that threatens job satisfaction is viewed critically by GPs and—from an occupational health point of view—has to be seen as critical in terms of work-related psychosocial stress, even more so if it increases an already high workload. The SCAs were seen as a potential resource if they allowed GPs to focus on patient contact, thus reducing unfavored work tasks and allowing more time for favored work content. Some authors envision the physician-patient relationship to transform fundamentally through patients’ use of SCAs [78]. GPs in this study appear to be protective of the physician-patient relationship. This study shows that GPs currently see the SCA as another means of diversifying patients’ access to knowledge, rather than as a challenge to the doctor-patient relationship or their professional identity as medical experts. Treating the source of information as secondary and focusing on direct interaction with the patient can also be read as a resource for maintaining control in a network of increasing numbers of nonhuman participants. In this way, GPs also protect important pillars of their job satisfaction: the physician-patient relationship, their medical and interpersonal expertise, and the lead in the diagnostic process.

### Strengths and Limitations

This study focuses on the perspective of GPs, although practice assistants also play an important role in general practice [79]. Initially, we had aimed to conduct 10 interviews with GPs that represent heterogeneous lived experiences in general practice [28]. Finding interview partners proved to be a challenge. GPs are known to have very high workloads and long working hours, so it is generally difficult to involve them in research studies [37,38,80]. The situation was exacerbated by the fact that we were looking for interview partners at the peak of the COVID-19 pandemic due to the funding period. We used various common strategies to reach out to GPs and eventually succeeded in finding 8 interview partners. The interviews were conducted between December 2020 and February 2022. We do not see striking differences between earlier and later interviews, probably also due to the fact that no COVID-19–related symptom checkers were used in Germany which might have introduced symptom checkers to a wider population. The sample only includes White GPs despite attempts to reach GPs of color and Black GPs. In addition to the aforementioned challenges, we assume unfitting sampling strategies and too homogeneous professional networks of the researchers as further reasons. It remains unclear, which impact race would have had on the perceptions of and experiences with the SCA. Within the given sample, the interview partners represent a variety of contexts and experiences and the data are rich enough to include shared patterns and conflicting perspectives [81]. Our results create resonance [40] with existing literature but also expand it. On the one hand, in terms of the issues raised by GPs in this study, we see strong parallels with the discourse on SCAs that we developed in our corresponding scoping review [36]. On the other hand, our data suggest that neither techno-utopian nor techno-dystopian visions of the literature are a dominant perceived reality in general practice in Germany. Instead, practitioners operating in a health care system of increasing scarcity display an attitude of techno-pragmatism in their daily work.

### Conclusions

Some of the current scientific literature on symptom checkers presents rather techno-utopian visions of symptom checkers as a means to make health care more humane by supporting health care professionals and patients [58,82], democratizing access to knowledge and expertise [12,18], and relieving a burdened health care system [15,20,21]. Others emphasize techno-dystopian visions of a technology that outperforms or replaces humans [11,83] and becomes an uncontrollable participant in health care systems [16]. Our results show that symptom checkers are a “proximate future” [47] rather than a lived experience among GPs in Germany. The German Federal Association of Statutory Health Insurance Physicians established the so-called Patient-Navi [84], which shows similarities to symptom checkers, and some health insurance agents are working on their own symptom checkers. Our results help to understand the context in which these technologies might enter and to identify possible long-term effects in the future. Given the fact that the main challenges for GPs and patients can be found on the macro level of the health care system, this study also highlights that singular technological solutions do not solve complex societal issues, but can at best be one means in a plethora of means taken.

## Appendix

Multimedia Appendix 1 Interview guide.

## Abbreviations

AI: artificial intelligence
GP: general practitioner
SCA: symptom checker app
