# Supplementary material for: The Doctors, Their Patients, and the Symptom Checker App: Qualitative Interview Study With General Practitioners in Germany
Source: JMIR Hum Factors. 2024 Nov 18;11:e57360. doi: 10.2196/57360 (PMC11612597; doi:10.2196/57360)
Supplement: Multimedia Appendix 1 [file humanfactors_v11i1e57360_app1.docx]

**Introduction**

*About the interviewer*

My name is ...

I am [job title] and work as a research assistant at IASV.

*About the project*

You have already received the information about our project and the interview process. Before the interview starts, I will briefly explain the content and the process of the interview. The interview is a part of a project "Symptom checkers based on artificial intelligence - a multi-perspective analysis of ethical, legal and social implications" funded by the German Federal Ministry of Education and Research. In our subproject we conduct the interviews with the GPs.

*Anonymity.* [Notes on encryption pure and information on who has access to the data.]

Please send the consent form and payment data to us soon.

*Consent*. Your participation is voluntary and you can withdraw at any time. Do you agree? Do you have any further questions? If further questions arise during the interview or if you did not understand something, please do not hesitate to ask.

*Together with interview partner: Standardized query of personal data (voluntary)*

| **Introductory question (approx. 5 minutes)**  *What do you enjoy in particular about your work as a* *general practitioner?* | | |
| --- | --- | --- |
| ***Checklist of content aspects*** | ***Important questions*** | ***Optional questions*** |
| ***Clientele*** | *What kind of clientele do you have in your practice?*  *Where do you see your focus in diagnostics?* |  |

*We are aware that you have a very varied daily routine and that patient care is an extensive process. However, I will focus my questions primarily on the process of diagnosis.*

| ***Guiding question 1: 'Self-diagnosis' by patients (approx. 20 minutes)***  *Patients come to you with complaints and symptoms. Some patients already have their own assumptions and may have already obtained an assessment from a third party or may have done research. Do you have a typical example from your practice?* | | |
| --- | --- | --- |
| ***Checklist of content aspects*** | ***Important questions*** | ***Optional questions*** |
| **Impacts**  Work processes, Work content  Challenge for general practitioners | What impact does it have on your work?  To what extent do patients talk about how they came to conclusions for their assessment? | In the literature, the term “self-diagnosis” is often used in this context. What does it mean for you as a general practitioner when your patients try to make the diagnoses themselves?  What challenges do you see in dealing with such patients?  How do you proceed when a “self-diagnosis” and your diagnosis don't match?  How do you deal with patients who refuse to be dissuaded from their “self-diagnosis”?  We have the impression from other interviews that patients go to the general practitioner either too early or too late. In your opinion, what is the right time to come to you? |
| ***Own internet use*** | Experiences with Dr. Google: Do your patients address Google searches?  What do you observe in patients who use Google? | For which parts of your work do you search the internet or use digital applications yourself?  Do you use internet research for diagnosis? |
| ***Digitalization*** | What is your opinion on the digitization of the GP practice? | Apps in GP practice (sleep, weight loss; diabetes management)? |

| ***Guiding question 2: ADA app (approx. 20 minutes )***  **Introduction of the app**  There are already apps for smartphones, the so-called symptom checkers.  These apps are designed to support patients with regard to their symptoms: what illness they may be suffering from. And to help them decide what to do next (for example, whether they should go to a doctor with their symptoms or not).  I'll explain how such apps work with the ADA app as an example: After entering age, gender, and personal risk factors, the app asks about current symptoms: starting with a complaint, it asks more in-depth questions. The app then makes suggestions about the possible causes of these complaints. These suggestions are prioritized by algorithms supported by artificial intelligence and then visually displayed for the patient. For each prioritization, a recommendation is issued (e.g., visit the emergency room, consult a doctor, or treat the condition independently at home).  *Have you encountered such self-diagnostic apps?*  ***Scenario I: General practitioners with experience***  *What are your previous experiences and touch points on this topic?* | | |
| --- | --- | --- |
| ***Checklist of content aspects*** | ***Important questions*** | ***Optional questions*** |
| **Changes**  Work content/ Diagnosis  Work processes  Communication/  relationship | *What advantages and disadvantages do you see for the Symptom Checkers when used in a GP practice?* | In the literature, the app is sometimes described as a form of self-anamnesis and sometimes as a form of self-diagnosis. What does it do from your point of view?  In your experience, what is different about SCA compared to otherwise pre-informed patients?  What effect did this “self-diagnosis/self-anamnesis [adopt wording of interviewees]” have on your work?  Are patients who use such apps or “Dr. Google” the more labor-intensive patients for you?  Some people see apps like ADA as empowering patients, especially in the early detection of diseases. How do you see this?  How do you see this app as a path toward more shared decision making between physicians and patients?  How did you assess benefits for yourself? |
| ***Scenario II: General practitioners without experience***  *You have no experience with such apps for patients. What is your general opinion on the possibilities of such SCAs?* | | |
| ***Checklist of content aspects*** | ***Important questions*** | ***Optional questions*** |
| **Expected changes**  Work content/  Diagnosis  Work processes  Communication/  relationship | *What advantages and disadvantages do you expect from Symptom Checkers when used in your GP practice?*  ***[do you have a concrete situation/example in mind?]*** | In the literature, the app is sometimes described as a form of self-anamnesis and sometimes as a form of self-diagnosis. What do you think the app will do?  What will be different about SCA compared to otherwise pre-informed patients?  What impact will this 'self-diagnosis/self-anamnesis [adopt wording of interviewees]' have on your work?  How do you assess, are the patients who use such apps or even Dr. Google the more labor-intensive patients for you?  Some people see apps like ADA as empowering patients, especially in the early detection of diseases. How do you see this?  How do you see these apps as a path toward more shared decision making between doctors and patients?  How did you assess benefits for yourself?  How would these apps support patients? |

| ***View (about 10 minutes)***  *What are your thoughts on the future of “self-diagnostic” apps in primary care?* | | |
| --- | --- | --- |
| ***Checklist of content aspects*** | ***Specific questions*** | ***Optional questions*** |
| ***Future of the SCA*** | How will this develop further?  How could this be well integrated into primary care so that it really benefits primary care physicians and patients? | There are symptom checkers for Covid-19 nowadays. How do you see such developments? |

| ***Final question (approx. 5 minutes)***  *What hasn't been mentioned yet that is important to you?* |
| --- |
